# Supplementary material for: Patient-Reported Outcome Measures for Evaluating Body Awareness: A Systematic Review Using the COSMIN Methodology
Source: Healthcare (Basel). 2025 Dec 12;13(24):3270. doi: 10.3390/healthcare13243270 (PMC12732759; doi:10.3390/healthcare13243270)
Supplement: Supplementary file 1 [file healthcare-13-03270-s001.zip › Table S5.pdf]

Table S5. Results from the psychometric outcomes extracted from the selected studies.

	Language	Structural validity		Internal consistency		Reliability		Hypotheses testing for construct validity				Criterion validity		Responsiveness		Measurement error	
		n		n		n		n	**Convergent**	**Divergent**	**Known**	n		n		n	
Awareness-Body-Chart (ABC)	*Original* German (Austria) (Danner et al., 2017)	106	KMO = 0.82; Total variance explained = 85.4%	106	ABC: α = 0.96<br>Body parts: α = 0.64-0.97	106	Total r = 0.96<br>Body parts r = 0.71-0.90	106	KEKS=0.66								
Body Awareness Rating Questionnaire (BARQ)	*Original* Norwegian (Dragesund et al., 2010)	300	Total variance explained = 46.9%	300	Total α = 0.78<br>Function α = 0.85<br>Mood α = 0.76<br>Feelings α = 0.79<br>Awareness α = 0.69												
	Norwegian (Tove et al., 2012)			50	Function α = 0.92<br>Mood α = 0.90<br>Feelings α = 0.93<br>Awareness α = 0.88	50	Function ICC = 0.85<br>Mood ICC = 0.83<br>Feelings ICC = 0.87<br>Awareness ICC = 0.79	50	Function: SF-36=0.08,0.53<br>Mood: SF-36=-0.50,0.42<br>Feelings: SF-36=-0.02,0.39<br>Awareness: SF-36=-0.10,0.15	Function: TAS=-0.16,0.08<br>Mood: TAS=0.06,0.26<br>Feelings: TAS=-0.35,-0.25<br>Awareness: TAS=-0.23,-0.17				50	Function d=0.53 (p<0.001)<br>Mood p=0.26)<br>Feelings p=0.35)<br>Awareness d=0.82 (p<0.001)		Function SDC = 0.85<br>Mood SDC = 0.76<br>Feelings SDC = 0.79<br>Awareness SDC = 0.69
	Turkish (Demirel et al., 2020)			99+101	Function α = 0.65-0.90<br>Mood α = 0.82-0.88<br>Feelings α = 0.74-0.96<br>Awareness α = 0.81-0.92	99+101	Function ICC = 0.65-0.90<br>Mood ICC = 0.82-0.87<br>Feelings ICC = 0.74-0.96<br>Awareness ICC = 0.82-0.92	99	Function: VAS=-0.08 SF-36=0.34,0.50<br>Mood: VAS=0.17 SF-36=0.11,0.26<br>Feelings: VAS=0.05 SF-36=0.22,0.38<br>Awareness: VAS= 0.07 SF-36=0.10,0.40	Function: ODI=-0.34 TAS=-0.27<br>Mood: ODI=-0.09 TAS=-0.33<br>Feelings: ODI=-0.10 TAS=-0.39<br>Awareness: ODI=-0.30 TAS=-0.21							
Revised Body Awareness Rating Questionnaire (BARQ-R)	*Original* Norwegian (Dragesund et al., 2018)	127	X^2=13.6 (p=0.004) Fit residual= −2.0 to 0.5	127	PSI=0.76	48	ICC=0.83									48	SDC=6.26

			Item difficulty=1.3, −1.3														
	English (USA)(Carpentier et al., 2024)	623	X^2 =205.7 (p<0.001)<br>Fit residual= −0.5 to 0.7	623	PSI=0.72												
Body Awareness Questionnaire (BAQ)	*Original* English (USA)(Shields et al., 1989)	794	Total variance explained = 46%	794	TOTAL α =0.82	70	ICC=0.80	794	BES=0.14, 0.34;<br>SCI=0.05, 0.25;<br>BCI=0.07, 0.66;<br>RSES=0.02, 0.16	EPI=-0.15,0.35;<br>PILL=-0.01,0.28	Aerobics instructors vs no-instructors (p=0.03)						
	Sweden(Lööf et al., 2013)	120	CFI = 0.68-0.75;<br>RMSEA = 0.07-0.09;<br>SRMR = 0.08	120 +120	Students:<br>TOTAL α=0.74<br>DBC α=0.52<br>PBR α=0.63<br>SC α=0.65<br>OI α=0.25<br><br>Rheumatic patients:<br>TOTAL α=0.90<br>DBC α=0.71<br>PBR α=0.79<br>SC α=0.89<br>OI α=0.62												
	German (Germany)(Cramer et al., 2018)	512	KMO = 0.86; Total variance explained = 50.7%	512	TOTAL α = 0.84	?	ICC=0.86	512	BRQ=-0.18, 0.45;<br>DBII=0.00, 0.16;<br>CPSC=0.27					T0:202<br>T1:168	p<0.001		
	Turkish (Karaca & Bayar, 2021)	180	KMO = 0.86; Total variance explained = 66%	180	α = 0.91	90	ICC=0.83	180	SCS=0.80	BPS=-0.75							
	Turkish(Unal et al., 2021)			180	α = 0.76	180	ICC=0.78	180	RSES=0.18								
	Spanish (Sánchez-Sánchez et al., 2025)	277	X^2 =142.0 (p<0.001)<br>RMSEA = 0.06	277	TOTAL α =0.82<br><br>MPE α =0.80<br><br>PBR α =0.72			281	MPE: BAS-2=0.32<br>NSSS-S=0.06<br>SCS-S=0.27<br><br>PBR: BAS-2=0.12<br>NSSS-S=-0.05<br>SCS-S=0.02		Mediation experience vs no experience (p < 0.001, d=0.59)						
	French (Carre et al., 2024)	610	X^2 =316.7 (p<0.001)<br>CFI = 0.98<br>RMSEA = 0.04	610	α =0.82<br><br>ω=0.83			412-198	BVAQ= -0.14<br>TAS= -0.24								
Body Perception Questionnaire-Short form (BPQ-SF)	*Original* Spanish (Spain)(Cabrera et al., 2018)	465	CFI = 0.95<br>RMSEA = 0.11	465	BA ω=0.92<br><br>SUP ω=0.89<br><br>SUB ω=0.77	53	BA ICC = 0.99<br><br>SUP ICC = 0.97<br><br>SUB ICC = 0.96	465	BA: SRI=0.57;<br>SSAS=0.51<br><br>SUP: SRI=0.65;<br>SSAS=0.46<br><br>SUB: SRI=0.58;<br>SSAS=0.42								

	Original English (USA)(Cabrera et al., 2018)	855*	CFI = 0.94-0.98<br>RMSEA = 0.02-0.03	855	BA ω=0.92-0.96<br><br>SUP ω = 0.88-0.94<br><br>SUB ω = 0.78-0.87												
	Italian(Cerritelli et al., 2021)	493	CFI = 0.98<br>RMSEA = 0.03	493	BA ω=0.92<br><br>SUP ω=0.88<br><br>SUB ω=0.86												
	Chinese (China)(Wang et al., 2020)	688	CFI = 0.90<br>RMSEA = 0.06	688	BPQ-SF: r=0.94<br><br>BA: r=0.90<br><br>SUP: r = 0.88<br><br>SUB: r = 0.85	83	BPQ-SF: r=0.78<br><br>BA: r=0.72<br><br>SUP: r=0.71<br><br>SUB: r=0.74	688	BPQ-SF:<br>SCL-90-s=0.49:<br>SDS-SOM=0.32<br><br>BA:<br>SCL-90-s=0.42;<br>SDS-SOM=0.29<br><br>SUP:<br>SCL-90-s=0.48;<br>SDS-SOM=0.27<br><br>SUB:<br>SCL-90-s=0.37;<br>SDS-SOM=0.24	BPQ-SF:<br>SDS-PSY=0.19<br><br>BA: SDS-PSY=0.16<br><br>SUP: SDS-PSY=0.19<br><br>SUB: SDS-PSY=0.11							
	Persian (Najari et al., 2024)	748	RMSEA = 0.046<br>CFI = 0.973<br>TLI = 0.958	748	BPQ-SF ω=0.94<br><br>BA ω=0.90<br><br>SUP ω = 0.89<br><br>SUB ω = 0.74				BPQ-SF:<br>DASS=0.57;<br>SCL90=0.60<br><br>BA:<br>DASS=0.58;<br>SCL90=0.64<br><br>SUP:<br>DASS=0.49;<br>SCL90=0.54<br><br>SUB:<br>DASS=0.64;<br>SCL90=0.69								
Body Perception Questionnaire-Very short form (BPQ-VSF)	*Original* Spanish (Spain)(Cabrera et al., 2018)			465	ω = 0.86	53	ICC = 0.97	465	SRI=0.55;<br>SSAS=0.48			465	BPQ-SF r=0.94				
	Original English (USA)(Cabrera et al., 2018)			855	ω = 0.83-0.91												
	Chinese (China)(Wang et al., 2020)			688	ω = 0.84	83	r = 0.68	688	SCL-90-s=0.42;<br>SDS-SOM=0.28	SDS-PSY=0.18							
Multidimensional Assessment of Interoceptive Awareness (MAIA)	*Original* English (USA)(Mehling et al., 2012)	325	CFI = 0.88<br>RMSEA = 0.06<br>SRMR = 0.05	325	N: α=0.69<br><br>ND: α=0.66<br><br>NW: α=0.67			325	N: FFMQ=0.19, 0.53;<br>PBCS=0.40;<br>EACS=0.25;<br>BRQ=0.31, 0.46	N: ASI-PC=0.19;<br>PCS=-0.27, -0.20;<br>SBC-BD=-0.30;<br>DERS=-0.36, -0.22;<br>STAI-T=-0.33	Subscales: Students vs Professors (4/8) p=0.001-0.38						

					AR: α=0.87<br><br>EA: α=0.82<br><br>SR: α=0.83<br><br>BL: α=0.82<br><br>T: α=0.79				ND: FFMQ=0.17, 0.41; PBCS=0.20; EACS=0.15; BRQ=0.31, 0.38<br><br>NW: FFMQ=0.13, 0.47; PBCS=0.01; EACS=0.11; BRQ=0.19, 0.35<br><br>AR: FFMQ=0.28, 0.55; PBCS=0.43; EACS=0.33; BRQ=0.33, 0.48<br><br>EA: FFMQ=0.14, 0.50; PBCS=0.32; EACS=0.34; BRQ=0.24, 0.48<br><br>SR: FFMQ=0.18, 0.46; PBCS=0.29; EACS=0.27; BRQ=0.27, 0.45<br><br>BL: FFMQ=0.21, 0.50; PBCS=0.33; EACS=0.43; BRQ=0.33, 0.64<br><br>T: FFMQ=0.27, 0.42; PBCS=0.28; EACS=0.21; BRQ=0.40, 0.53	ND: ASI-PC=0.18; PCS=-0.17, -0.24; SBC-BD=-0.32; DERS=-0.24, -0.33; STAI-T=-0.35<br><br>NW: ASI-PC=0.36; PCS=-0.40, -0.46; SBC-BD=-0.27; DERS=-0.11, -0.44; STAI-T=-0.46<br><br>AR: ASI-PC=0.31; PCS=-0.19, -0.30; SBC-BD=-0.41; DERS=-0.23, -0.41; STAI-T=-0.38<br><br>EA: ASI-PC=0.12; PCS=-0.15, -0.25; SBC-BD=-0.33; DERS=-0.13, -0.44; STAI-T=-0.19<br><br>SR: ASI-PC=0.20; PCS=-0.28, -0.34; SBC-BD=-0.27; DERS=-0.20, -0.38; STAI-T=-0.46<br><br>BL: ASI-PC=0.18; PCS=-0.24, -0.34; SBC-BD=-0.34; DERS=-0.21, -0.54; STAI-T=-0.29<br><br>T: AASI-PC=0.23; PCS=-0.24, -0.31; SBC-BD=-0.39; DERS=-0.22, -0.39; STAI-T=-0.46							
	Chinese (Taiwan)(Lin et al., 2017)	294	CFI = 0.95<br>RMSEA = 0.07<br>SRMR = 0.09	294	N: α=0.76<br><br>ND: α=0.58<br><br>NW: α=0.46<br><br>AR: α=0.85<br><br>EA: α=0.88<br><br>SR: α=0.81<br><br>BL: α=0.87<br><br>T: α=0.86	38	N: ICC=0.68<br><br>ND: ICC=0.60<br><br>NW: ICC=0.73<br><br>AR: ICC=0.85<br><br>EA: ICC=0.84<br><br>SR: ICC=0.82<br><br>BL: ICC=0.83	294			Subscales: Less vs highly experienced (6/8) p=0.001-0.56						

							T: ICC=0.83										
	German (Germany)(Bornemann et al., 2015)			1076	N: α=0.76<br><br>ND: α=0.56<br><br>NW: α=0.65<br><br>AR: α=0.89<br><br>EA: α=0.86<br><br>SR: α=0.84<br><br>BL: α=0.84<br><br>T: α=0.86	80	N: r=0.73<br><br>ND: r=0.66<br><br>NW: r=0.76<br><br>AR: r=0.72<br><br>EA: r=0.77<br><br>SR: r=0.78<br><br>BL: r=0.78<br><br>T: r=0.79	1076	N: FFMQ=-0.05, 0.51; PBCS=0.42<br><br>ND: FFMQ=-0.06, 0.22; PBCS=0.17<br><br>NW: FFMQ=-0.11, 0.39; PBCS=-0.05<br><br>AR: FFMQ=-0.04, 0.42; PBCS=0.22<br><br>EA: FFMQ=-0.08, 0.56; PBCS=0.43<br><br>SR: FFMQ=-0.06, 0.41; PBCS=0.26<br><br>BL: FFMQ=-0.04, 0.55; PBCS=0.37<br><br>T: FFMQ=-0.24, 0.43; PBCS=0.20	MAIA-N: STAI-T=0.03<br><br>MAIA-ND: STAI-T=-0.11<br><br>MAIA-NW: STAI-T=-0.43<br><br>MAIA-AR: STAI-T=-0.18<br><br>MAIA-EA: STAI-T=0.06<br><br>MAIA-SR: STAI-T=-0.24<br><br>MAIA-BL: STAI-T=-0.05<br><br>MAIA-T: STAI-T=-0.44				232	N p=0.44<br>ND p=0.12<br>NW p=0.18<br>AR d=0.54(p<0.001)<br>EA p=0.04<br>SR d=0.72 (p<0.001)<br>BL d=0.40 (p<0.001)<br>T p<0.001		
	Greek(Vinni et al., 2021)	107	70.6% total variance explained	107	N: α=0.80<br><br>ND: α=0.66<br><br>NW: α=0.64<br><br>AR: α=0.85<br><br>EA: α=0.88<br><br>SR: α=0.84<br><br>BL: α=0.82<br><br>T: α=0.65												
	Japanese(Shoji et al., 2018)	390	55.3% total variance explained	390	N: α=0.74<br><br>ND: α=0.67<br><br>AR: α=0.87<br><br>EA: α=0.85<br><br>BL: α=0.84<br><br>T: α=0.83			251	N: FFMQ=0.19<br><br>ND: FFMQ=0.18<br><br>AR: FFMQ=0.42<br><br>EA: FFMQ=0.20<br><br>BL: FFMQ=0.44<br><br>T: FFMQ=0.54	N: STAI-T=0.02; PCS=0.12; DERS=0.02<br><br>ND: STAI-T=-0.23; PCS=-0.20; DERS=-0.24<br><br>AR: STAI-T=-0.29; PCS=-0.08; DERS=-0.27<br><br>EA: STAI-T=-0.09; PCS=0.04; DERS=-0.03<br><br>BL: STAI-T=-0.34; PCS=-0.01; DERS=-0.24							

										T: FFMQ= STAI-T=-0.46; PCS=-0.06; DERS=-0.32							
	Japanese(Fujino, 2019)	268	6-factors<br>CFI = 0.98<br>RMSEA = 0.07<br>SRMR = 0.06	268	N: α=0.78<br><br>ND: α=0.72<br><br>AR: α=0.87<br><br>EA: α=0.84<br><br>BL: α=0.82<br><br>T: α=0.80	78	N: ICC=0.85<br><br>ND: ICC=0.76<br><br>AR: ICC=0.85<br><br>EA: ICC=0.82<br><br>BL: ICC=0.82<br><br>T: ICC=0.78	268	N: BAS=0.35, 0.49<br><br>ND: BAS=-0.06, 0.10<br><br>AR: BAS=0.35, 0.44<br><br>EA: BAS=0.28, 0.25<br><br>BL: BAS=0.35, 0.33<br><br>T: BAS=0.29, 0.44								
	Italian(Calì et al., 2015)	321	CFI = 0.97<br>RMSEA = 0.02<br>SRMR = 0.05	321	N: α=0.68<br><br>ND: α=0.53<br><br>NW: α=0.59<br><br>AR: α=0.75<br><br>EA: α=0.79<br><br>SR: α=0.75<br><br>BL: α=0.74<br><br>T: α=0.80			321		N: ESS=0.60<br><br>ND: ESS=-0.09<br><br>NW: ESS=-0.33<br><br>AR: ESS=-0.22<br><br>EA: ESS=0.21<br><br>SR: ESS=-0.09<br><br>BL: ESS=0.12<br><br>T: ESS=-0.28							
	Spanish (Chile)(Valenzuela-Moguillansky & Reyes-Reyes, 2015)	470	67.2% total variance explained<br>CFI = 0.92<br>RMSEA = 0.05<br>SRMR = 0.05	470	MAIA: α=0.90<br><br>N: α=0.63<br><br>ND: α=0.48<br><br>NW: α=0.40<br><br>AR: α=0.86<br><br>EA: α=0.81<br><br>SR: α=0.85<br><br>BL: α=0.83<br><br>T: α=0.85												
	Spanish (Colombia)(Montoya-Hurtado et al., 2023)	202	(No specific data available)	202	α = 0.90; ω = 0.96												
	English (USA)(Brown et al., 2017)	376	62.0% total variance explained<br>CFI = 0.88	376	N: α=0.76<br><br>ND: α=0.67<br><br>NW: α=0.62			376		N: STAI-T=-0.10; TAS=-0.20, -0.12; DERS=-0.35, -0.02							

					AR: α=0.91<br><br>EA: α=0.84<br><br>SR: α=0.89<br><br>BL: α=0.89<br><br>T: α=0.92					ND: STAI-T=-0.17; TAS=-0.15; DERS=-0.16, -0.06<br><br>NW: STAI-T=-0.22; TAS=-0.27, -0.16; DERS=-0.31, 0.00<br><br>AR: STAI-T=-0.41; TAS=-0.38, -0.35; DERS=-0.52, -0.25<br><br>EA: STAI-T=-0.25; TAS=-0.33, -0.22; DERS=-0.42, -0.12<br><br>SR: STAI-T=-0.49; TAS=-0.40, -0.38; DERS=-0.51, -0.30<br><br>BL: STAI-T=-0.39; TAS=--0.38, -0.35; DERS=-0.51, -0.28<br><br>T: STAI-T=-0.66; TAS=-0.52, -0.49; DERS=-0.55, -0.38							
	Hungarian (Ferentzi et al., 2021)	612 + 1497	CFI = 0.98 RMSEA = 0.06 SRMR = 0.05	612 + 1497	N: α=0.69<br><br>ND: α=0.59<br><br>NW: α=0.74<br><br>AR: α=0.88<br><br>EA: α=0.85<br><br>SR: α=0.85<br><br>BL: α=0.87<br><br>T: α=0.88			612 + 1497	N: MAAS=0.18; BAQ=0.44; SSAS=0.13; HEXACO=-0.05, 0.24<br><br>ND: MAAS=0.20; BAQ=0.12; SSAS=0.07; HEXACO=0.00, 0.07<br><br>NW: MAAS=0.18; BAQ=0.14; SSAS=-0.33; HEXACO=-0.49, 0.27<br><br>AR: MAAS=0.25; BAQ=0.47; SSAS=0.02; HEXACO=-0.29, 0.34<br><br>EA: MAAS=0.14; BAQ=0.54; SSAS=0.13; HEXACO=0.01, 0.23<br><br>SR: MAAS=0.28; BAQ=0.48; SSAS=-0.10; HEXACO=-0.32, 0.37<br><br>BL: MAAS=0.21; BAQ=0.48; SSAS=0.06; HEXACO=-0.11, 0.26	N: STAI-T=-0.14; PCS=-0.09<br><br>ND: STAI-T=-0.14; PCS=-0.05<br><br>NW: STAI-T=-0.32; PCS=-0.62<br><br>AR: STAI-T=-0.31; PCS=-0.25<br><br>EA: STAI-T=-0.07; PCS=0.01<br><br>SR: STAI-T=-0.40; PCS=-0.26<br><br>BL: STAI-T=-0.21; PCS=-0.05<br><br>T: STAI-T=-0.49; PCS=-0.25							

									T: MAAS=0.29; BAQ=0.38; SSAS=-0.09; HEXACO=-0.30, 0.47								
	Lithuanian(Baranauskas et al., 2016)	386	CFI = 0.85<br>RMSEA = 0.07<br>SRMR = 0.07	386	N: α=0.54<br>ND: α=0.40<br>NW: α=0.63<br>AR: α=0.79<br>EA: α=0.73<br>SR: α=0.79<br>BL: α=0.81<br>T: α=0.82												
	Malay(Todd et al., 2020)	438	53.0% total variance explained<br>CFI = 0.94<br>RMSEA = 0.06<br>SRMR = 0.04	438	AR: ω=0.89<br>EA: ω=0.88<br>T: ω=0.88												
	Portuguese(Machorrinho et al., 2019)	204 + 286	60.0% total variance explained<br>CFI = 0.82<br>RMSEA = 0.07<br>SRMR = 0.07	286	N: α=0.61<br>ND: α=0.81<br>NW: α=0.74<br>AR: α=0.86<br>EA: α=0.80<br>SR: α=0.87<br>T: α=0.81	20	N: ICC=0.57<br>ND: ICC=0.52<br>NW: ICC=0.69<br>AR: ICC=0.59<br>EA: ICC=0.82<br>SR: ICC=0.82<br>T: ICC=0.83	40	N: FFMQ=-0.13, 0.27<br>ND: FFMQ=-0.32, 0.09<br>NW: FFMQ=-0.44, -0.02<br>AR: FFMQ=-0.15, 0.48<br>EA: FFMQ=-0.18, 0.46<br>SR: FFMQ=-0.24, 0.48<br>T: FFMQ=-0.42, 0.54	N: STAI-T=0.38; STAI-A=0.30<br>ND: STAI-T=0.03; STAI-A=-0.14<br>NW: STAI-T=-0.09; STAI-A=0.04<br>AR: STAI-T=0.47; STAI-A=0.45<br>EA: STAI-T=0.45; STAI-A=0.16<br>SR: STAI-T=0.34; STAI-A=0.37<br>T: STAI-T=-0.20; STAI-A=0.13							
Multidimensional Assessment of Interoceptive Awareness Version 2 (MAIA-2)	*Original* English (USA)(Mehling et al., 2018)	545	CFI = 0.86<br>RMSEA = 0.05<br>SRMR = 0.06	1090	N: α=0.64<br>ND: α=0.74<br>NW: α=0.67<br>AR: α=0.83<br>EA: α=0.79<br>SR: α=0.79<br>BL: α=0.80												

					T: α=0.83												
	English (USA) (Chapman & Stewart, 2024)	710	RMSEA = 0.046<br>SRMR = 0.063<br>CFI = 0.917	710	N: α=0.72<br><br>ND: α=0.71<br><br>NW: α=0.81<br><br>AR: α=0.86<br><br>EA: α=0.79<br><br>SR: α=0.82<br><br>BL: α=0.84<br><br>T: α=0.88	66	N: r=0.70<br><br>ND: r=0.67<br><br>NW: r=0.66<br><br>AR: r=0.73<br><br>EA: r=0.79<br><br>SR: r=0.73<br><br>BL: r=0.75<br><br>T: r=0.73										
	French(Da Costa Silva et al., 2022)	154	CFI = 0.95<br>RMSEA = 0.11<br>SRMR = 0.10	308	MAIA-2: α=0.90<br><br>N: α=0.77, ω=0.76<br><br>ND: α=0.71, ω=0.57<br><br>NW: α=0.84, ω=0.84<br><br>AR: α=0.89, ω=0.89<br><br>EA: α=0.85, ω=0.86<br><br>SR: α=0.85, ω=0.85<br><br>BL: α=0.77, ω=0.77<br><br>T: α=0.84, ω=0.83	308	MAIA-2: ICC=0.81<br><br>N: ICC=0.69<br><br>ND: ICC=0.66<br><br>NW: ICC=0.72<br><br>AR: ICC=0.63<br><br>EA: ICC=0.74<br><br>SR: ICC=0.74<br><br>BL: ICC=0.73<br><br>T: ICC=0.82	308	MAIA-2: PAS=0.60; FMI=0.64<br><br>N: PAS=0.45; FMI=0.38<br><br>ND: PAS=0.21; FMI=0.16<br><br>NW: PAS=-0.06; FMI=-0.07<br><br>AR: PAS=0.54; FMI=0.54<br><br>EA: PAS=0.39; FMI=0.35<br><br>SR: PAS=0.43; FMI=0.58<br><br>BL: PAS=0.45; FMI=0.48<br><br>T: PAS=0.40; FMI=0.53	MAIA-2: TAS=-0.50; BFI=-0.28,0.25<br><br>N: TAS=-0.30; BFI=-0.03,0.21<br><br>ND: TAS=-0.27; BFI=-0.03,0.14<br><br>NW: TAS=0.20; BFI=-0.24,0.09<br><br>AR: TAS=-0.37; BFI=-0.26,0.24<br><br>EA: TAS=-0.30; BFI=-0.03,0.26<br><br>SR: TAS=-0.37; BFI=-0.30,0.22<br><br>BL: TAS=-0.33; BFI=-0.07,0.27<br><br>T: TAS=-0.42; BFI=-0.44,0.24							
	Arabic (Lebanon) (Fekih-Romdhane et al., 2023)	359	RMSEA = 0.06<br>SRMR = 0.05<br>CFI = 0.90	359	N: ω=0.86<br><br>ND: ω=0.90<br><br>NW: ω=0.82<br><br>AR: ω=0.93<br><br>EA: ω=0.90<br><br>SR: ω=0.90<br><br>BL: ω=0.88<br><br>T: ω=0.90			359	N: IES-2-RHSC=0.26<br><br>ND: IES-2-RHSC=-0.19<br><br>NW: IES-2-RHSC=0.02<br><br>AR: IES-2-RHSC=0.31<br><br>EA: IES-2-RHSC=0.23<br><br>SR: IES-2-RHSC=0.28								

									BL: IES-2-RHSC=0.35<br><br>T: IES-2-RHSC=0.37							
	Chinese (China) (Teng et al., 2022)	627	RMSEA = 0.06<br>SRMR = 0.08<br>CFI = 0.89	627	MAIA-2: α=0.82<br><br>ND: α=0.80<br><br>NW: α=0.65<br><br>AR: α=0.82<br><br>EA: α=0.81<br><br>SR: α=0.74<br><br>BL: α=0.76<br><br>T: α=0.83			627	ND: FFMQ=-0.22, 0.25<br><br>NW: FFMQ=-0.16, 0.17<br><br>AR: FFMQ=0.28, 0.46<br><br>EA: FFMQ=-0.29, 0.51<br><br>SR: FFMQ=-0.23, 0.46<br><br>BL: FFMQ=-0.34, 0.53<br><br>T: FFMQ=-0.14, 0.35	ND: STAI-T=-0.10<br><br>NW: STAI-T=-0.14<br><br>AR: STAI-T=-0.19<br><br>EA: STAI-T=-0.08<br><br>SR: STAI-T=-0.25<br><br>BL: STAI-T=-0.10<br><br>T: STAI-T=-0.25							
	Dutch (Netherlands) (Scheffers et al., 2024)	527	RMSEA = 0.05<br>SRMR = 0.05<br>CFI = 0.86	1054	N: α=0.67, ω=0.67<br><br>ND: α=0.80, ω=0.81<br><br>NW: α=0.78, ω=0.78<br><br>AR: α=0.86, ω=0.86<br><br>EA: α=0.80, ω=0.79<br><br>SR: α=0.81, ω=0.82<br><br>BL: α=0.82, ω=0.82<br><br>T: α=0.88, ω=0.89	109	N: ICC=0.79<br><br>ND: ICC=0.67<br><br>NW: ICC=0.69<br><br>AR: ICC=0.74<br><br>EA: ICC=0.73<br><br>SR: ICC=0.73<br><br>BL: ICC=0.68<br><br>T: ICC=0.70										
	Norwegian (Fiskum et al., 2023)	306	RMSEA = 0.05<br>CFI = 0.95	306	N: α=0.78, ω=0.75<br><br>ND: α=0.89, ω=0.87<br><br>NW: α=0.82, ω=0.78<br><br>AR: α=0.87, ω=0.85	306		306	MAIA-2: COOP=0.09, 0.46<br><br>N: COOP=0.04, 0.18<br><br>ND: COOP=0.11, 0.28<br><br>NW: COOP=0.01, 0.34<br><br>AR: COOP=0.04, 0.31								

					EA: α=0.83, ω=0.81<br><br>SR: α=0.87, ω=0.86<br><br>BL: α=0.89, ω=0.93<br><br>T: α=0.95, ω=0.93				EA: COOP=0.02, 0.18<br><br>SR: COOP=0.07, 0.38<br><br>BL: COOP=0.01, 0.23<br><br>T: COOP=0.07, 0.56								
	Persian (Melhi et al., 2021)	475	RMSEA = 0.05<br>SRMR = 0.06<br>CFI = 0.91	475	N: α=0.58<br><br>ND: α=0.84<br><br>NW: α=0.79<br><br>AR: α=0.88<br><br>EA: α=0.83<br><br>SR: α=0.85<br><br>BL: α=0.87<br><br>T: α=0.87			475	N: MAAS=0.24<br><br>ND: MAAS=0.38<br><br>NW: MAAS=0.29<br><br>AR: MAAS=0.35<br><br>EA: MAAS=0.21<br><br>SR: MAAS=0.18<br><br>BL: MAAS=0.33<br><br>T: MAAS=0.32								
	Spanish (Peru) (Vivas-Rivas et al., 2025)	414	RMSEA = 0.06<br>SRMR = 0.06<br>CFI = 0.93	414	N: α=0.86, ω=0.84<br><br>ND: α=0.90, ω=0.86<br><br>NW: α=0.66, ω=0.63<br><br>AR: α=0.89, ω=0.88<br><br>EA: α=0.88, ω=0.88<br><br>SR: α=0.86, ω=0.86<br><br>BL: α=0.87, ω=0.85<br><br>T: α=0.89, ω=0.88												
Brief Multidimensional Assessment of Interoceptive Awareness Version 2 (Brief MAIA-2)	Polish(Rogowska et al., 2023)	323	KMO = 0.92;<br>Bifactor model RMSEA = 0.05;<br>SRMR = 0.04; CFI = 0.94		Brief MAIA-2: α=0.84, ω=0.88<br><br>N: α=0.67, ω=0.68<br><br>ND: α=0.78, ω=0.79												

					NW: α=81, ω=0.82<br><br>AR: α=0.86, ω=0.86<br><br>EA: α=0.86, ω=0.86<br><br>SR: α=0.85, ω=0.85<br><br>BL: α=0.79, ω=0.79<br><br>T: α=0.89, ω=0.90												
Physical Activity Body Experiences Questionnaire (PABE)	*Original* English (USA)(Menzel et al., 2019)	606	Total variance explained = 71.5%	606	BMR: α=0.90<br><br>AYB: α=0.85<br><br>APS: α=0.86<br><br>APL: α=0.77	58	Subscales: r=?-0.72	606	PABE: BAQ=0.56; BRQ=0.64; MBSRQ-AE=0.45; RSES=0.42<br><br>BMR: BAQ=0.48; BRQ=0.57; MBSRQ-AE=0.36; RSES=0.31<br><br>AYB: BAQ=0.66; BRQ=0.65; MBSRQ-AE=0.56; RSES=0.49<br><br>APC: BAQ=0.37; BRQ=0.53; MBSRQ-AE=0.29; RSES=0.34<br><br>APL: BAQ=0.35; BRQ=0.34; MBSRQ-AE=0.26; RSES=0.32	PABE: OBCS=-0.22, -0.16; SOQ =-0.10; EDE-q=-0.21<br><br>BMR: OBCS=-0.11, -0.19; SOQ =-0.12; EDE-q=-0.14<br><br>AYB: OBCS=-0.34, -0.18; SOQ=-0.09, EDE-q=-0.34<br><br>APC: OBCS=-0.13, -0.05; SOQ=-0.05; EDE-q=-0.08<br><br>APL: OBCS=-0.20, -0.12; SOQ=-0.05; EDE-q=-0.13							
Physical Body Experiences Questionnaire Simplified for Active Aging (PBE-QAG)	*Original* Italian(Cossu et al., 2018)	106	CFI = 0.989, RMSEA = 0.076	106	PBE-QAG: α=0.89<br><br>BMR: α=0.80<br><br>AYB: α=0.81<br><br>APS: α=0.83<br><br>APL: α=0.65												
	English (USA)(Deng et al., 2023)	566	Person mean location = -1.77±1.22 logits														
Scale of Body Connection (SBC)	*Original*	291	CFI = 0.96; RMSEA =	291	BA: α=0.83												

	English (USA)(Price & Thompson, 2007)		0.05; SRMR = 0.07		BD: α=0.78												
	Portuguese (Portugal) (Neves et al., 2017)	445	CFI = 0.74; RMSEA = 0.09	445				445	BA: AD-CDS=0.12<br><br>BD: GBD=0.38; AD-CDS=0.45; PD-CDS=0.31								
	Portuguese (Portugal) (Price et al., 2017)	909	BA: CFI = 0.97 RMSEA = 0.05<br><br>BD: CFI = 0.97; RMSEA = 0.06	909	BA: α=0.86<br><br>BD: α=0.71												
	Italian(Price et al., 2017)	576	BA: CFI = 0.95; RMSEA = 0.05<br><br>BD: CFI = 0.96; RMSEA = 0.05	576	BA: α=0.82<br><br>BD: α=0.64												
	French(Price et al., 2017)	198	BA: CFI = 0.90; RMSEA = 0.08<br><br>BD: CFI = 0.91; RMSEA = 0.10	198	BA: α=0.83<br><br>BD: α=0.75												
	Dutch (Netherlands)(Price et al., 2017)	434	BA: CFI = 0.92; RMSEA = 0.05<br><br>BD: CFI = 0.94; RMSEA = 0.05	434	BA: α=0.72<br><br>BD: α=0.63												
	English (USA)(Price et al., 2017)	909*	BA: CFI = 0.95-0.97; RMSEA = 0.04-0.06<br><br>BD: CFI = 0.93-0.99; RMSEA = 0.02-0.08	909*	BA: α=0.84-0.86<br><br>BD: α=0.76-0.81												
	Hebrew (Israel)(Price et al., 2017)	608	BA: CFI = 0.96; RMSEA = 0.06	608	BA: α=0.86												

	English (USA)(Cheng et al., 2022)			287*	BA: α=0.78-0.93<br><br>BD: α=0.74-0.87			99*	BA: FFMQ=0.48<br><br>BD: FFMQ=0.64	BA: DERS=0.45<br><br>BD: DERS=0.46-0.64		*		287*	BA d = 0.14-0.47 (p=0.001-0.66)<br><br>BD d = 0.08-0.86 (p=0.01-0.62)		
	French(Cheng et al., 2022)			12	BA: α=0.53<br><br>BD: α=0.70			12	BA: FFMQ=0.45<br><br>BD: FFMQ=0.63					12	BA d = 1.23 (p<0.001)<br><br>BD d = 0.79 (p=0.01)		
	Dutch (Netherlands) (Cheng et al., 2022)			214*	BA: α=0.80-0.82<br><br>BD: α=0.55-0.69									214*	BA d = 0.67-0.71 (p=0.001-0.002)<br><br>BD d = 0.54-0.58 (p=0.001-0.02)		
	German(Cheng et al., 2022)			582*	BA: α=0.73-0.85<br><br>BD: α=0.68-0.79									582*	BA d = 0.12-0.47 (p=0.001-0.58)<br><br>BD d = 0.02-0.12 (p=0.02-0.96)		
	Italian(Morganti et al., 2020)	576	CFI = 0.84; RMSEA = 0.06; SRMR = 0.07-0.08	576	BA: α=0.82<br><br>BD: α=0.65			576	BA: IRI=0.05-0.31<br><br>BD: IRI=0.02-0.31								
	Spanish (Spain)(del C Quezada-Berumen et al., 2014)	578	CFI = 0.96; RMSEA = 0.05; SRMR = 0.07	578	BA: α=0.86<br><br>BD: α=0.62	67	BA: r=0.67<br><br>BD: r=0.76	578	BA: FFMQ=0.22-0.65; DASS-21=0.07-0.22<br><br>BD: FFMQ=0.07-0.54; DASS-21=0.40-0.42								

AD-CDS, Appearance-based Distraction subscale of Cognitive Distortion Scales; APS, Awareness of Physical Skills Subscale; APL, Awareness of Physical Limits Subscale; ASI-PC, Anxiety Sensitivity Index – Physical Concern; AYB, Accepting Your Body Subscale; BA, Body Awareness Subscale; BAS, Body Awareness Scale; BCI, Body-Conciousness Inventory; BD, Bodily Dissociation Subscale; BES, Body Esteem Scale; BFI, Big Five Inventory; BMR, Body-mind Relationship Subscale; BPS, Body Perception Scale; BRQ, Body Responsiveness Questionnaire; BVAQ-B, Bermond-Vorts Alexithymia Questionnaire; COOP, Dartmouth Coop Functional Health Assessment/World Organization of National Colleges, Academies and Academic Association of General Practitioners; CPSC, Conscious Presence and Self Control; DASS-21, Depression Anxiety Stress Scale 21; DBII, Dresden Body Image Inventory; DERS, Difficulties in Emotion Regulation Scale; DBC, Detecting bodily changes subscale; EACS, Emotional Approach Coping Scales – Emotional Processing; EDE-Q, Eating Disorder Examination Questionnaire; EPI, Eysenck Personality Inventory; ESS, Emotional Susceptibility Scale; FFMQ, Five Facet Mindfulness Questionnaire; FMI, Freiburg Mindfulness Inventory; GBD, General Body Dissatisfaction; HEXACO, HEXACO Personality Inventory; IES-2-RHSC, The intuitive eating scale-2-Reliance on Hunger and Satiety Cues; IRI, Interpersonal Reactivity Index; KEKS, Short Validated Questionnaire of Body Self-awareness; MAAS, Mindful Attention Awareness Scale; MBSRQ-AE, Appearance Evaluation subscale of the Multidimensional Body-Self Relations Questionnaire; MPE, Monitoring physical energy levels; OBC, Objectified Body Consciousness Scale; OI, Onset of illness subscale; PAS, Postural Awareness Scale; PCS, Pain Catastrophizing Scale; PBCS, Private Body Consciousness Scale; PBR, Predicting body responses subscale; PD-CDS, Performance-based Distraction subscales of Cognitive Distortion Scales; PILL, Pennebaker Inventory of Limbic Languidness; RSES, Rosenberg Self-Esteem Scale; SC, Sleep-wake cycle subscale; SCI, Self-Consciousness Inventory; SCL-90-S, Symptom Checklist 90 Somatization Subscale; SCS, Self-Consciousness Scale; SDS-PSY, Self-Rating Depression Scale Psychological Symptoms Subscale; SDS-SOM, Self-Rating Depression Scale Somatic Symptoms Subscale; SOQ, Self-objectification Questionnaire; SRI, Stress Reactivity Index; SSAS, Somatosensory Amplification Scale; STAI-A, Trait Anxiety Inventory; STAI-T, State Anxiety Inventory; SUB, Subdiaphragmatic Reactivity Subscale; SUP, Supradiaphragmatic Reactivity Subscale; TAS, Toronto Alexithymia Scale; PSI, Person Separation Index; SDC, Standard Detectable Change; PGIC, Patient Global Impression of Change.

*, Summation of several different studies by the authors of the study; ???, No info.
