# Supplementary material for: Patient-Reported Outcome Measures for Evaluating Body Awareness: A Systematic Review Using the COSMIN Methodology
Source: Healthcare (Basel). 2025 Dec 12;13(24):3270. doi: 10.3390/healthcare13243270 (PMC12732759; doi:10.3390/healthcare13243270)
Supplement: Supplementary file 1 [file healthcare-13-03270-s001.zip › Table S1.pdf]

Table S1. Search strings and number of results in each database. 2024/12/17

Search string	PubMed	Results
**#1**	("body awareness" or "movement awareness" or "body mind interventions" or "mind body intervention" or "body consciousness" or "movement quality")	**2,228**
**#2**	(instrumentation OR methods OR 'validation studies' OR 'comparative study' OR 'psychometrics' OR 'psychometr*' OR 'clinimetr*' OR 'outcome assessment (health care)' OR 'outcome assessment' OR 'outcome measure*' OR 'observer variation' OR 'health status indicators' OR 'reproducibility of results' OR 'reproducib*' OR 'discriminant analysis' OR 'reliab*' OR 'unreliab*' OR 'valid*' OR 'coefficient' OR 'homogeneity' OR 'homogeneous' OR 'internal consistency' OR ('cronbach*' AND ('alpha' OR 'alphas')) OR ('item' AND ('correlation*' OR 'selection*' OR 'reduction*')) OR 'agreement' OR 'precision' OR 'imprecision' OR 'precise values' OR 'test retest' OR ('test' AND 'retest') OR ('reliab*' AND ('test' OR 'retest')) OR 'stability' OR 'interrater' OR 'inter-rater' OR 'intrarater' OR 'intra-rater' OR 'intertester' OR 'inter-tester' OR 'intratester' OR 'intra-tester' OR 'interobserver' OR 'inter-observer' OR 'intraobserver' OR 'intertechnician' OR 'inter-technician' OR 'intratechnician' OR 'intra-technician' OR 'interexaminer' OR 'inter-examiner' OR 'intraexaminer' OR 'intra-examiner' OR 'interassay' OR 'inter-assay' OR 'intraassay' OR 'intra-assay' OR 'interindividual' OR 'inter-individual' OR 'intraindividual' OR 'intra-individual' OR 'interparticipant' OR 'inter-participant' OR 'intraparticipant' OR 'intra-participant' OR 'kappa' OR 'kappas' OR 'repeatab*' OR (('replicab*' OR 'repeated') AND ('measure' OR 'measures' OR 'findings' OR 'result' OR 'results' OR 'test' OR 'tests')) OR 'generaliza*' OR 'generalisa*' OR 'concordance' OR ('intraclass' AND 'correlation*') OR 'discriminative' OR 'known group' OR 'factor analysis' OR 'factor analyses' OR 'dimension*' OR 'subscale*' OR ('multitrait' AND 'scaling' AND ('analysis' OR 'analyses')) OR 'item discriminant' OR 'interscale correlation*' OR 'error' OR 'errors' OR 'individual variability' OR ('variability' AND ('analysis' OR 'values')) OR ('uncertainty' AND ('measurement' OR 'measuring')) OR 'standard error of measurement' OR 'sensitiv*' OR 'responsive*' OR (('minimal' OR 'minimally' OR 'clinical' OR 'clinically') AND ('important' OR 'significant' OR 'detectable') AND ('change' OR 'difference')) OR ('small*' AND ('real' OR 'detectable') AND ('change' OR 'difference')) OR 'meaningful change' OR 'ceiling effect' OR 'floor effect' OR 'item response model' OR 'irt' OR 'rasch' OR 'differential item functioning' OR 'dif' OR 'computer adaptive testing' OR 'item bank' OR 'cross-cultural equivalence')	**12,735,527**
**#3**	(addresses OR biography OR 'case reports' OR comment OR directory OR editorial OR festschrift OR interview OR lectures OR 'legal cases' OR legislation OR letter OR news OR 'newspaper article' OR 'patient education handout' OR 'popular works' OR congresses OR 'consensus development conference' OR 'consensus development conference, nih' OR 'practice guideline') NOT (animals NOT humans)	**6,099,448**
**#4**	**(#1 AND #2) NOT #3**	**1,411**
	**Scopus**	

**#1**	TITLE-ABS-KEY("body awareness" OR "movement awareness" OR "body mind interventions" OR "mind body intervention" OR "body consciousness" OR "movement quality")	**4,281**
**#2**	TITLE-ABS-KEY (instrumentation OR methods OR "validation studies" OR "comparative study" OR psychometrics OR psychometr* OR clinimetr* OR "outcome assessment (health care)" OR "outcome assessment" OR "outcome measure*" OR "observer variation" OR "health status indicators" OR "reproducibility of results" OR reproducib* OR "discriminant analysis" OR reliab* OR unreliab* OR valid* OR coefficient OR homogeneity OR homogeneous OR "internal consistency" OR (cronbach* AND (alpha OR alphas)) OR (item AND (correlation* OR selection* OR reduction*)) OR agreement OR precision OR imprecision OR "precise values" OR "test retest" OR (test AND retest) OR (reliab* AND (test OR retest)) OR stability OR interrater OR inter-rater OR intrarater OR intra-rater OR intertester OR inter-tester OR intratester OR intra-tester OR interobserver OR inter-observer OR intraobserver OR intertechnician OR inter-technician OR intratechnician OR intra-technician OR interexaminer OR inter-examiner OR intraexaminer OR intra-examiner OR interassay OR inter-assay OR intraassay OR intra-assay OR interindividual OR inter-individual OR intraindividual OR intra-individual OR interparticipant OR inter-participant OR intraparticipant OR intra-participant OR kappa OR kappas OR repeatab* OR ((replicab* OR repeated) AND (measure OR measures OR findings OR result OR results OR test OR tests)) OR generaliza* OR generalisa* OR concordance OR (intraclass AND correlation*) OR discriminative OR "known group" OR "factor analysis" OR "factor analyses" OR dimension* OR subscale* OR (multitrait AND scaling AND (analysis OR analyses)) OR "item discriminant" OR "interscale correlation*" OR error OR errors OR "individual variability" OR (variability AND (analysis OR values)) OR (uncertainty AND (measurement OR measuring)) OR "standard error of measurement" OR sensitiv* OR responsive* OR ((minimal OR minimally OR clinical OR clinically) AND (important OR significant OR detectable) AND (change OR difference)) OR ((small* AND (real OR detectable) AND (change OR difference))) OR "meaningful change" OR "ceiling effect" OR "floor effect" OR "item response model" OR irt OR rasch OR "differential item functioning" OR dif OR "computer adaptive testing" OR "item bank" OR "cross-cultural equivalence")	**40,781,612**
**#3**	TITLE-ABS-KEY (addresses OR biography OR "case reports" OR comment OR directory OR editorial OR festschrift OR interview OR lectures OR "legal cases" OR legislation OR letter OR news OR "newspaper article" OR "patient education handout" OR "popular works" OR congresses OR "consensus development conference" OR "consensus development conference, nih" OR "practice guideline")	**10,284,624**
**#4**	**(#1 AND #2) NOT #3**	**2,271**

PsycInfo		
**#1**	("body awareness" or "movement awareness" or "body mind interventions" or "mind body intervention" or "body consciousness" or "movement quality")	**3,993**
**#2**	(instrumentation OR methods OR 'validation studies' OR 'comparative study' OR 'psychometrics' OR 'psychometr*' OR 'clinimetr*' OR 'outcome assessment (health care)' OR 'outcome assessment' OR 'outcome measure*' OR 'observer variation' OR 'health status indicators' OR 'reproducibility of results' OR 'reproducib*' OR 'discriminant analysis' OR 'reliab*' OR 'unreliab*' OR 'valid*' OR 'coefficient' OR 'homogeneity' OR 'homogeneous' OR 'internal consistency' OR ('cronbach*' AND ('alpha' OR 'alphas')) OR ('item' AND ('correlation*' OR 'selection*' OR 'reduction*')) OR 'agreement' OR 'precision' OR 'imprecision' OR 'precise values' OR 'test retest' OR ('test' AND 'retest') OR ('reliab*' AND ('test' OR 'retest')) OR 'stability' OR 'interrater' OR 'inter-rater' OR 'intrarater' OR 'intra-rater' OR 'intertester' OR 'inter-tester' OR 'intratester' OR 'intra-tester' OR 'interobserver' OR 'inter-observer' OR 'intraobserver' OR 'intertechnician' OR 'inter-technician' OR 'intratechnician' OR 'intra-technician' OR 'interexaminer' OR 'inter-examiner' OR 'intraexaminer' OR 'intra-examiner' OR 'interassay' OR 'inter-assay' OR 'intraassay' OR 'intra-assay' OR 'interindividual' OR 'inter-individual' OR 'intraindividual' OR 'intra-individual' OR 'interparticipant' OR 'inter-participant' OR 'intraparticipant' OR 'intra-participant' OR 'kappa' OR 'kappas' OR 'repeatab*' OR (('replicab*' OR 'repeated') AND ('measure' OR 'measures' OR 'findings' OR 'result' OR 'results' OR 'test' OR 'tests')) OR 'generaliza*' OR 'generalisa*' OR 'concordance' OR ('intraclass' AND 'correlation*') OR 'discriminative' OR 'known group' OR 'factor analysis' OR 'factor analyses' OR 'dimension*' OR 'subscale*' OR ('multitrait' AND 'scaling' AND ('analysis' OR 'analyses')) OR 'item discriminant' OR 'interscale correlation*' OR 'error' OR 'errors' OR 'individual variability' OR ('variability' AND ('analysis' OR 'values')) OR ('uncertainty' AND ('measurement' OR 'measuring')) OR 'standard error of measurement' OR 'sensitiv*' OR 'responsive*' OR (('minimal' OR 'minimally' OR 'clinical' OR 'clinically') AND ('important' OR 'significant' OR 'detectable') AND ('change' OR 'difference')) OR ('small*' AND ('real' OR 'detectable') AND ('change' OR 'difference')) OR 'meaningful change' OR 'ceiling effect' OR 'floor effect' OR 'item response model' OR 'irt' OR 'rasch' OR 'differential item functioning' OR 'dif' OR 'computer adaptive testing' OR 'item bank' OR 'cross-cultural equivalence')	**2,650,204**
**#3**	(addresses OR biography OR 'case reports' OR comment OR directory OR editorial OR festschrift OR interview OR lectures OR 'legal cases' OR legislation OR letter OR news OR 'newspaper article' OR 'patient education handout' OR 'popular works' OR congresses OR 'consensus development conference' OR 'consensus development conference, nih' OR 'practice guideline') NOT (animals NOT humans)	**1,150,329**
**#4**	**(#1 AND #2) NOT #3**	**1,568**
