# Supplementary material for: Patient-Reported Outcome Measures for Evaluating Body Awareness: A Systematic Review Using the COSMIN Methodology
Source: Healthcare (Basel). 2025 Dec 12;13(24):3270. doi: 10.3390/healthcare13243270 (PMC12732759; doi:10.3390/healthcare13243270)
Supplement: Supplementary file 1 [file healthcare-13-03270-s001.zip › Table S2.pdf]

Table S2. Definitions and decision criteria of the measurement properties according to the Cosmin guideline (COSMIN Tool Version 2.0).

**Measurement property**	**Definition**	**Criteria for good measurement properties**
Content validity	The degree to which the content of a PROM is an adequate reflection of the construct to be measured.	+: Included items are relevant for the construct, target population, and context of use, and response options and recall period are appropriate<br>AND<br>No key concepts are missing<br>AND<br>PROM items and response options are appropriately worded and PROM instructions, items and response options understood by the population of interest as intended
		?: Not enough information
		-: Included items are not relevant for the construct or target population<br>OR<br>Key concepts are missing<br>OR<br>PROM items and response options are not appropriately worded or not understood by the population of interest as intended
Structural validity	The degree to which the scores of a PROM are an adequate reflection of the dimensionality of the construct to be measured.	+:<br><u>Classic factor tests:</u><br>EFA: factor loadings of each item on its factor ≥0.30<br>AND<br>Maximum 10% of the items have factor loadings ≥0.30 on multiple factors<br>AND<br>Explained variance ≥50% and structure is in line with the theory about the construct to be measured OR results on scree plot or Kaiser criterion (Eigenvalues >1) are in line with the theory about the construct to be measured<br><br>CFA: CFI or TLI or comparable measure >0.95 OR RMSEA <0.06 OR SRMR <0.08<br><br><u>Item response theory/Rasch:</u><br>No violation of unidimensionality: CFI or TLI or comparable measure >0.95 OR RMSEA <0.06 OR SRMR <0.08<br>AND<br>No violation of local independence: residual correlations among the items after controlling for dominant factor <0.20 OR Q3s <0.37

		AND<br>No violation of monotonicity: adequate looking graphs OR item scalability >0.30<br>AND<br>Adequate model fit:<br>IRT: $\chi 2$ >0.01<br>Rasch: infit and outfit mean squares ≥0.5 and ≤1.5 OR Z-standardized values >-2 and <2
		?: Not enough information reported
		-: Criteria for '+' not met
Internal consistency	The degree of the interrelatedness among the items.	+: At least low evidence for sufficient unidimensionality<br>AND<br>Cronbach's alpha ≥0.70
		?: Criteria for "at least low evidence for sufficient unidimensionality" not met<br>OR<br>Evidence for insufficient unidimensionality<br>OR<br>Not enough information reported
		-: At least low quality evidence for sufficient unidimensionality<br>AND<br>Cronbach's alpha <0.70
Cross-cultural validity	The degree to which the performance of the items on a translated or culturally adapted PROM are adequate reflection of the performance of the items of the original version of the PROM.	+: No important differences found between group factors (such as age, gender, language) in multiple group factor analysis OR<br>no important differential item functioning for group factors (McFadden's R2 <0.02)
		?: Not enough information reported
		-: Important differences between group factors OR differential item functioning was found
Reliability	The extent to which scores for patients who have not changed are the same for repeated measurement under several conditions.	+: ICC or (weighted) kappa ≥0.70 or Pearson/Spearman correlation ≥0.70
		?: Not enough information reported
		-: ICC or (weighted) kappa <0.70 or Pearson/Spearman correlation <0.70
Measurement error	The systematic and random error of a patient's score that is not attributed to true changes in the construct to be measured.	+: SDC or LoA <MIC
		?: MIC not defined OR not enough information reported
		-: SDC or LoA > MIC

Criterion validity	The degree to which the scores of a PROM are an adequate reflection of a 'gold standard'.	+: Correlation with gold standard ≥0.70 OR AUC ≥0.70
		?: Not enough information reported
		-: Correlation with gold standard <0.70 OR AUC <0.70
Hypotheses testing for construct validity	The degree to which the scores of a PROM are consistent with hypotheses based on the assumption that the PROM validly measures the construct to be measured.	+: ≥75% of the results is in accordance with predefined hypotheses
		?: No relevant results were found
		-: ≥75% of the results deviates from predefined hypotheses
Responsiveness	The ability of a PROM to detect change over time in the construct to be measures.	+: ≥75% of the results is in accordance with predefined hypotheses OR AUC ≥0.70
		?: No relevant results were found
		-: ≥75% of the results deviates from predefined hypotheses OR AUC <0.70

AUC, area under the curve; CFA, confirmatory factor analysis; CFI = comparative fit index; EFA, exploratory factor analysis; ICC = intraclass correlation coefficient; LoA, limits of agreement; MIC, minimal important change; PROM, patient reported outcome measure; RMSEA, Root Mean Square Error of Approximation; SDC, smallest detectable change; SRMR, Standardized Root Mean Residuals; TLI, Tucker-Lewis index.

Source: https://www.cosmin.nl/wp-content/uploads/COSMIN-Criteria-for-good-measurement-properties-version-2.0.pdf
