# Supplementary material for: Patient-Reported Outcome Measures for Evaluating Body Awareness: A Systematic Review Using the COSMIN Methodology
Source: Healthcare (Basel). 2025 Dec 12;13(24):3270. doi: 10.3390/healthcare13243270 (PMC12732759; doi:10.3390/healthcare13243270)
Supplement: Supplementary file 1 [file healthcare-13-03270-s001.zip › Table S3.pdf]

Table S3. Psychometric analysis of all the properties assessed in all the selected PROMs.

	**Language**	**Content validity**	**Cross-cultural translation process**	**Structural validity**	**Internal consistency**	**Reliability**	**Hypotheses testing for construct validity**			**Criterion validity**	**Responsiveness**	**Measurement error**
							**Convergent**	**Divergent**	**Known**			
Awareness-Body-Chart (ABC)	*Original*<br>German (Austria)(Danner et al., 2017)	Doubtful		Adequate (?)	Very good<br>Total (+)<br>Subscales (±)	Doubtful<br>Total (+)<br>Subscales (+)	Very good (+)					
Body Awareness Rating Questionnaire (BARQ)	*Original*<br>Norwegian (Dragesund et al., 2010)	Doubtful		Doubtful (-)	Doubtful (+)							
	Norwegian (Tove et al., 2012)		n.a.		Doubtful<br>Subscales (+)	Adequate<br>Subscales (+)	Adequate<br>Subscales (-)	Adequate<br>Subscales (-)				Adequate (?)
	Turkish (Demirel et al., 2020)		4/5		Doubtful<br>Subscales (±)	Inadequate<br>Subscales (+)	Adequate<br>Subscales (±)	Adequate<br>Subscales (+)				
Revised Body Awareness Rating Questionnaire (BARQ-R)	*Original*<br>Norwegian (Dragesund et al., 2018)			Very good (+)	Very good (+)	Very good (+)						Adequate (?)
	English (USA)(Carpentier et al., 2024)		No info	Very good (+)	Very good (+)							
Body Awareness Questionnaire (BAQ)	*Original*<br>English (USA)(Shields et al., 1989)	Doubtful		Doubtful (-)	Doubtful (+)	Very good (+)	Very good (-)	Adequate (-)	Adequate (+)			
	Sweden(Lööf et al., 2013)		2/5	Adequate (-)	Very good<br>Total (+)<br>Subscales (±)							
	German (Germany)(Cramer et al., 2018)		4/5	Adequate (+)	Doubtful<br>Total (+)<br>Subscales (±)	Doubtful (+)	Very good (-)				Adequate (+)	
	Turkish (Karaca & Bayar, 2021)		4/5	Doubtful (+)	Doubtful<br>Total (+)	Very good (+)	Very good (+)	Very good (+)				
	Turkish(Unal et al., 2021)		Based on a previous translation		Doubtful<br>Total (+)	Very good (+)	Adequate (-)					
	Spanish (Sánchez-Sánchez et al., 2025) (Sánchez-Sánchez et al., 2025)		2/5	Very good (-)	Very good<br>Total (+)<br>Subscales (+)		Adequate<br>Subscales (-)		Doubtful<br>Total (+)			
	French (Carre et al., 2024)		0/5	Very good (+)	Very good (+)		Adequate (-)					
Body Perception Questionnaire-Short form (BPQ-SF)	*Original*<br>Spanish (Spain)(Cabrera et al., 2018)	Doubtful		Very good (-)	Very good<br>Total (+)<br>Subscales (+)	Very good<br>Total (+)<br>Subscales (+)	Adequate<br>Total (+)<br>Subscales (+)					
	Original<br>English (USA)(Cabrera et al., 2018)	Doubtful		Very good (+)	Very good (+)<br>Total (+)<br>Subscales (+)							
	Italian(Cerritelli et al., 2021)		4/5	Doubtful (+)	Very good<br>Total (+)<br>Subscales (+)							
	Chinese (China)(Wang et al., 2020)		1/5	Doubtful (-)	Very good<br>Total (+)<br>Subscales (+)	Doubtful<br>Total (+)<br>Subscales (+)	Adequate<br>Total (+)<br>Subscales (-)	Adequate<br>Total (-)<br>Subscales (-)				
	Persian (Najari et al., 2024)		4/5	Very good (+)	Very good<br>Total (+)<br>Subscales (+)		Adequate<br>Total (+)<br>Subscales (+)					
Body Perception Questionnaire-Very short form (BPQ-VSF)	*Original*<br>Spanish (Spain)(Cabrera et al., 2018)	Doubtful			Very good (+)	Very good (+)	Adequate (+)			Very good (+)		
	Original	Doubtful			Very good (+)							

	English (USA)(Cabrera et al., 2018)											
	Chinese (China)(Wang et al., 2020)		Based on a previous translation		Very good (+)	Doubtful (-)	Adequate (-)	Adequate (-)				
Multidimensional Assessment of Interoceptive Awareness (MAIA)	*Original*<br>English (USA)(Mehling et al., 2012)	Doubtful		Very good (+)	Very good<br>Subscales (±)		Very good<br>Subscales (±)	Very good<br>Subscales (-)	Adequate<br>Subscales<br>(-)			
	Chinese (Taiwan)(Lin et al., 2017)		4/5	Doubtful (-)	Very good<br>Subscales (±)	Doubtful (±)			Adequate<br>Subscales<br>(+)			
	German (Germany)(Bornemann et al., 2014)		3/5		Very good<br>Subscales (±)	Inadequate (±)	Very good<br>Subscales (±)	Very good<br>Subscales (±)			Very good<br>Subscales (±)	
	Greek(Vinni et al., 2021)		4/5	Inadequate (?)	Very good<br>Subscales (±)							
	Japanese(Shoji et al., 2018)		3/5	Doubtful (?)	Very good<br>Subscales (±)		Very good<br>Subscales (±)	Very good<br>Subscales (-)				
	Japanese(Fujino, 2019)		N.a.	Doubtful (+)	Very good<br>Subscales (+)	Doubtful (+)	Very good<br>Subscales (±)					
	Italian(Calì et al., 2015)		3/5	Very good (+)	Very good<br>Subscales (±)			Doubtful<br>Subscales (±)				
	Spanish (Chile)(Valenzuela-Moguillansky & Reyes-Reyes, 2015)		4/5	Very good (+)	Very good<br>Total (+)<br>Subscales (±)							
	Spanish (Colombia)(Montoya-Hurtado et al., 2023)		Based on a previous translation	Adequate (?)	Inadequate<br>Total (+)							
	English (USA)(Brown et al., 2017)		Based on a previous translation	Doubtful (-)	Very good<br>Subscales (±)			Adequate<br>Subscales (±)				
	Hungarian (Ferentzi et al., 2021)		3/5	Very good (+)	Very good<br>Subscales (±)		Very good<br>Subscales (-)	Very good<br>Subscales (±)				
	Lithuanian(Baranauskas et al., 2016)		3/5	Inadequate (+)	Very good<br>Subscales (±)							
	Malay(Todd et al., 2020)		5/5	Very good (+)	Very good<br>Subscales (+)							
	Portuguese(Machorrinho et al., 2019)		3/5	Very good (+)	Very good<br>Subscales (±)	Doubtful (±)	Very good<br>Subscales (-)	Very good<br>Subscales (±)				
Multidimensional Assessment of Interoceptive Awareness Version 2 (MAIA-2)	*Original*<br>English (USA)(Mehling et al., 2018)	Doubtful		Very good (+)	Very good:<br>Subscales (±)							
	English (USA) (Chapman & Stewart, 2024)			Very good (+)	Very good:<br>Subscales (+)	Doubtful:<br>Subscales (±)						
	French(Da Costa Silva et al., 2022)		5/5	Very good (-)	Very good:<br>Total (+)<br>Subscales (+)	Doubtful:<br>Total (+)<br>Subscales (±)	Very good<br>Total (+)<br>Subscales (±)	Very good<br>Total (-)<br>Subscales (±)				
	Arabic (Lebanon)(Fekih-Romdhane et al., 2023)		3/5	Doubtful (+)	Very good:<br>Subscales (+)		Doubtful<br>Subscales (±)					
	Chinese (China)(Teng et al., 2022)		4/5	Very good (-)	Very good:<br>Total (+)<br>Subscales (±)		Very good<br>Subscales (±)	Very good<br>Subscales (-)				
	Dutch (Netherlands)(Scheffers et al., 2024)		5/5	Very good (+)	Very good:<br>Subscales (+)	Very good:<br>Subscales (±)						
	Norwegian(Fiskum et al., 2023)		2/5	Doubtful (+)	Very good:<br>Subscales (+)		Adequate<br>Total (-)<br>Subscales (-)					

	Persian(Melhi et al., 2021)		3/5	Doubtful (+)	Very good:<br>Subscales (±)		Very good<br>Subscales (±)					
	Spanish (Peru) (Vivas-Rivas et al., 2025)		2/5	Very good (+)	Very good:<br>Subscales (±)							
Brief Multidimensional Assessment of Interoceptive Awareness Version 2 (Brief MAIA-2)	Polish(Rogowska et al., 2023)		2/5	Doubtful (+)	Very good:<br>Total (+)<br>Subscales (±)							
Physical Activity Body Experiences Questionnaire (PABE)	*Original*<br>English (USA)(Menzel et al., 2019)	Doubtful		Adequate (?)	Very good<br>Total (+)<br>Subscales (+)	Doubtful<br>Subscales (±)	Very good<br>Total (+)<br>Subscales (+)	Very good<br>Total (-)<br>Subscales (-)				
Physical Body Experiences Questionnaire Simplified for Active Aging (PBE-QAG)	*Original*<br>Italian(Cossu et al., 2018)	Doubtful		Doubtful (-)	Very good<br>Total (+)<br>Subscales (±)							
	English (USA)(Deng et al., 2023)		Based on a previous translation	Very good (?)								
Scale of Body Connection (SBC)	*Original*<br>English (USA)(Price & Thompson, 2007)	Doubtful		Doubtful (+)	Very good<br>Subscales (+)							
	Portuguese (Portugal) (Neves et al., 2017)		2/5	Doubtful (-)	Very good<br>Subscales (±)		Adequate<br>Subscales (±)					
	Portuguese (Portugal) (Price et al., 2017)		No info	Doubtful<br>Subscales (+)	Very good<br>Subscales (+)							
	Italian(Price et al., 2017)		No info	Doubtful<br>Subscales (+)	Very good<br>Subscales (±)							
	French(Price et al., 2017)		No info	Doubtful<br>Subscales (-)	Very good<br>Subscales (+)							
	Dutch (Netherlands)(Price et al., 2017)		No info	Doubtful<br>Subscales (+)	Very good<br>Subscales (±)							
	English (USA)(Price et al., 2017)		No info	Doubtful<br>Subscales (?)	Very good<br>Subscales (+)							
	Hebrew (Israel)(Price et al., 2017)		No info	Doubtful<br>Subscales (+)	Very good<br>Subscales (+)							
	English (USA)(Cheng et al., 2022)		No info		Very good<br>Subscales (+)		Very good<br>Subscales (+)	Very good<br>Subscales (+)			Very good<br>Subscales (±)	
	French(Cheng et al., 2022)		No info		Very good<br>Subscales (-)		Doubtful<br>Subscales (+)				Very good<br>Subscales (+)	
	Dutch (Netherlands)(Cheng et al., 2022)		No info		Very good<br>Subscales (±)						Very good<br>Subscales (+)	
	German(Cheng et al., 2022)		No info		Very good<br>Subscales (±)						Very good<br>Subscales (±)	
	Italian(Morganti et al., 2020)		2/5	Very good (-)	Very good (+)		Adequate<br>Subscales (-)					
	Spanish (Spain)(del C Quezada-Berumen et al., 2014)		3/5	Doubtful (+)	Doubtful (+)	Doubtful (±)	Very good<br>Subscales (±)					

+, Sufficient; -, Insufficient; ±, Inconsistent; ?, Indeterminate; n.a., not applicable (translation process done in a previous study).
