# Supplementary material for: Patient-Reported Outcome Measures for Evaluating Body Awareness: A Systematic Review Using the COSMIN Methodology
Source: Healthcare (Basel). 2025 Dec 12;13(24):3270. doi: 10.3390/healthcare13243270 (PMC12732759; doi:10.3390/healthcare13243270)
Supplement: Supplementary file 1 [file healthcare-13-03270-s001.zip › Table S4.pdf]

Table S4. Analysis of the cross-cultural translation process.

PROM name and original language	Translated version	Stage I: Initial Translation		Stage II: Synthesis of The Translations		Stage III: Back Translation		Stage IV: Expert Committee		Stage V: Test of the Prefinal Version		Overall and comments
		**Description provided by the authors**	**Fulfilled criteria?**	**Description provided by the authors**	**Fulfilled criteria?**	**Description provided by the authors**	**Fulfilled criteria?**	**Description provided by the authors**	**Fulfilled criteria?**	**Description provided by the authors**	**Fulfilled criteria?**	
Body Awareness Rating Questionnaire (BARQ) Norwegian	Turkish (Demirel et al., 2020)	3 native Turkish speakers (AD, DO, OU) translated the original BARQ items English to Turkish.	Yes	A single Turkish translation was created from these 3 translations.	Yes	One person who is native English speaker and has not familiar with BARQ and also speaks Turkish fluently translated this Turkish questionnaire back to English.	No	The English questionnaire was compared with the original version, discrepancies were resolved by discussion and a provisional BARQ-T was created.	Yes	This provisional BARQ-T was applied to 10 native Turkish speaker patients with CLBP. The feedback on comprehensibility and completeness of the BARQ-T were assessed.	Yes	4/5
Revised Body Awareness Rating Questionnaire (BARQ-R) Norwegian	English (USA)(Carpentier et al., 2024)	-	-	-	-	-	-	-	-	-	-	No info
Body Awareness Questionnaire (BAQ) English (USA)	Sweden (Lööf et al., 2013)	3 of the authors (FS, HL & U-B J)	No	A preliminary Swedish version was developed by synthesis of the translations by these authors.	Yes	A four-member back-translation committee (EWH, FS, HL & U-B J)	No	The authorized bilingual translator then examined discrepancies between the original English version and the Swedish version.	Yes	-	-	2/5
	German (Germany) (Cramer et al., 2018)	Two German native speakers with intensive English language training and knowledge of the English-speaking culture (HC, RL)	Yes	Both translations were combined by the translators into a single consensus translation by discussion.	Yes	Two independent professional translators.	Yes	Concordance of the back-translated version and the original BAQ was discussed by the translators, the developer of the original instrument (SS) and an English native speaking.	Yes	-	-	4/5
	Turkish (Karaca & Bayar, 2021)	Four independent translators translated the questionnaire into Turkish.	Yes	-	-	The professors and field experts living in the United States have also been translated back to the original.	Yes	The translators synthesized the reverse translations. Finally, the translator committee determined the inconsistencies and differences.	Yes	30 students who had an undergraduate education in Muğla Sıtkı Koçman University Faculty of Health Sciences. Individuals were asked to evaluate the items that they had difficulty in understanding the scale, suitability to Turkish, readability,	Yes	4/5

										and item order between 1-4 points.		
	Turkish (Unal et al., 2021)	-	-	-	-	-	-	-	-	-	-	Based on a previous translation
	Spanish (Sánchez-Sánchez et al., 2025)	The original English version was translated into Spanish and back-translated into English by two independent bilingual translators (Spanish and English).	Yes	-	-	The original English version was translated into Spanish and back-translated into English by two independent bilingual translators (Spanish and English).	Yes	-	-	-	-	2/5
	French (Carre et al., 2024)	The French version was adapted using the translation and backtranslation process performed by a native English-speaking colleague to ensure its accuracy.	No	-	-	The French version was adapted using the translation and backtranslation process performed by a native English-speaking colleague to ensure its accuracy.	No	-	-	-	-	0/5
Body Perception Questionnaire-Short form (BPQ-SF) Spanish (Spain) and English (USA)	Italian (Cerritelli et al., 2021)	Two native Italian speakers fluent in English.	Yes	A common forward translated version was agreed upon by the two translators.	Yes	Native speakers who were fluent in Italian.	Yes	Expert committee including the translators, a linguistic expert, two osteopaths, and one epidemiologist.	Yes	-	-	4/5
	Chinese (China) (Wang et al., 2020)	Independent bilingual psychological major postgraduates	Yes	-	-	-	-	-	-	-	-	1/5
	Persian (Najari et al., 2024)	The BPQ-SF was translated by three psychologists, all fluent in Persian and English.	Yes	The Persian version was assessed by expert psychologist.	Yes	It was back-translated into English by two professional translators.	Yes	The two versions were compared regarding content and translation quality, and no fundamental difference was observed between the original and the back-translated version.	Yes	-	-	4/5
Body Perception Questionnaire-Very short form (BPQ-VSF) Chinese (China)	Chinese (China) (Wang et al., 2020)	-	-	-	-	-	-	-	-	-	-	Based on a previous translation
Multidimensional Assessment of Interoceptive Awareness (MAIA)	Chinese (Taiwan) (Lin et al., 2017)	Five native Chinese speakers who spoke English and were familiar with both cultures each	Yes	Discrepancies among their five translations were thoroughly discussed and amended to produce	Yes	Two individuals, an English-language college teacher trained in linguistics in Taiwan and a	Yes	-	-	Five teenage Chinese speakers and three senior nursing experts in a Taiwanese hospital	Yes	4/5

English (USA)				the most appropriate and adequate translation.		U.S.-based physician specializing in physical medicine and rehabilitation						
	German (Bornemann et al., 2015)	Two of the authors (Wolf E. Mehling and Boris Bornemann, both native German speakers and proficient in English), and a translation agency (Baker and Harrison, Munich, Germany)	Yes	Wolf E. Mehling and Boris Bornemann then compared the three translations, item by item, and, in the case of different translations, picked the wording that was most easily understandable and closest to the English version.	Yes	The final questionnaire was then sent to the agency and translated back into English by another independent translator.	No	The back-translation and the original English questionnaire were compared.	Yes	-	-	3/5
	Greek(Vinni et al., 2021)	Two independent translators who were native speaking Greek language	Yes	A third independent translator and Greek native-speaking reconciled the two forward translations to one single target language translation, creating a revised version of the questionnaire.	Yes	A native-speaking English language translator, who speaks Greek language fluently and had never read the original version of the questionnaire translated independently and separately the reconciled version of the questionnaire from Greek into English (backward translation).	No	A review of the back-translated and reconciled version of the questionnaire was performed by an evaluation committee comprised of three natives Greek-speaking experts	Yes	A pilot-testing of the first consensus of the questionnaire was performed, including cognitive debriefing interviews with 6 native Greek-speaking adults	Yes	4/5
	Japanese(Shoji et al., 2018)	Translation office (6 native Japanese experts).	Yes	Finally, comments made during every step of this translation process were discussed in an international expert panel including MS, BMH, WM and SO	Yes	-	-	-	-	MS conducted short cognitive interviews with all Japanese participants that completed the MAIA.	Yes	3/5
	Japanese(Fujino, 2019)	-	-	-	-	-	-	-	-	-	-	Based on a previous translation
	Italian(Calì et al., 2015)	Two by native Italian speakers proficient in English, and one by a native English speaker proficient in Italian.	Yes	The three resulting provisional Italian versions were then compared, item by item, by two of the authors (EA and GCo) and two other researchers.	Yes	An English native bilingual translator, who had a background in Psychology but who was not familiar with the construct, performed the back-translation into English.	No	Finally, differences between the original English version and the back-translation were identified and discussed with the first author of the original MAIA.	Yes	-	-	3/5

	Spanish (Chile)(Valenzuela-Moguillansky & Reyes-Reyes, 2015)	Three independent forward translations were made: two by bilingual Spanish native translators who didn't know the construct and one by a bilingual Spanish native person who was familiar with the construct.	Yes	The three versions were compared and, after consensus between the two translators and the project manager, a single document was drafted.	Yes	An English native bilingual translator, who was not familiar with the construct, performed the back-translation into English.	No	Divergences between the back-translation and the original English version were identified and discussed with the first author of the original scale. For the items where cross-language agreement could not be reached, Spanish sentences were reworded.	Yes	The cognitive interviews sample included thirteen people aged 21 to 72 (M = 42.8; SD = 15.6), with education level from high school to graduate school.	Yes	4/5
	Spanish (Colombia) (Montoya-Hurtado et al., 2023)	-	-	-	-	-	-	-	-	-	-	Based on a previous translation
	English (USA) (Brown et al., 2017)	-	-	-	-	-	-	-	-	-	-	Based on a previous translation
	Hungarian(Ferentzi et al., 2021)	The English version has been translated by two experts independently.	Yes	After they agreed on the final version	Yes	An independent third person backtranslated MAIA to English.	No	A native speaker compared the two English versions and made some suggestions	Yes	-	-	3/5
	Lithuanian(Baranauskas et al., 2016)	Independently by the researchers, i. e. MB and AG, and also by the translation office.	Yes	Researchers had a discussion with the purpose to choose the most proper forms of items.	Yes	-	-	-	-	32 students of kinesiotherapy at Vilnius University.	Yes	3/5
	Malay(Todd et al., 2020)	Forward-translated from English to Malay by an informed and an uninformed translator.	Yes	Two translations were examined by a third independent and blind translator, who resolved discrepancies between the translations and produced a synthesised forward-translation.	Yes	In a third stage, two new independent and blind translators back-translated the synthesised translation into English.	Yes	In a fourth stage, the forward- and back-translations were examined by a bilingual committee comprising all aforementioned translators, a methodologist, and the final four authors of the present study.	Yes	42 Malayan women.	Yes	5/5
	Portuguese(Machorrinho et al., 2019)	Three native Portuguese speakers, fluent in English and familiar with the concepts of interoception and mindfulness, independently translated the 38 items of the English MAIA version into Portuguese	Yes	The three Portuguese versions were discussed by these experts to ensure the conceptual equivalence of the items.	Yes	-	-	-	-	The consensus version was then cognitively tested in a 1-hour focus group of seven university students, led by two moderators.	Yes	3/5

Multidimensional Assessment of Interoceptive Awareness Version 2 (MAIA-2)<br>English (USA)	French(Da Costa Silva et al., 2022)	The questionnaires were translated by native French-speakers [one psychologist (CB), one researcher in the field of neuroscience (AD), and one medical doctor (CV)].	Yes	The translated questionnaires were back translated by three English speakers totally blind to the original version [one American student in neuroscience (BR), one professional translator (ES), and one naive French speaker with fluency in English (CGV)]	Yes	The translated questionnaires were back translated by three English speakers totally blind to the original version [one American student in neuroscience (BR), one professional translator (ES), and one naive French speaker with fluency in English (CGV)]	Yes	A final harmonization meeting involving translators of the two steps procedure (CB, AD, CV, ES, and CGV) as well as a student in clinical psychology (LDCS), was held in order to come to satisfactory formulations and validate the translation process.	Yes	A “field test” was performed with a group of 20 participants to determine whether the translated items of the PAS and MAIA-2 retained the same meaning as the original items.	Yes	5/5
	Arabic (Lebanon)(Fekih-Romdhane et al., 2023)	The English version was translated to Arabic by a Lebanese translator who was completely unrelated to the study.	No	The initial and translated English versions were compared to detect and later eliminate any inconsistencies by a committee composed of the research team and the two translators.	Yes	Afterwards, a Lebanese psychologist with a full working proficiency in English, translated the Arabic version back to English.	No	The initial and translated English versions were compared to detect and later eliminate any inconsistencies by a committee composed of the research team and the two translators	Yes	A pilot study was conducted on 20 persons before the start of the official data collection.	Yes	3/5
	Chinese (China)(Teng et al., 2022)	Three native Chinese bilingual speakers, two did not know the construct and one was familiar with the construct, completed the forward-translation into Chinese independently.	Yes	After comparing the three translated versions, we discussed them with one native Chinese bilingual professor and formed a forward-translated version.	Yes	A bilingual overseas doctoral student, who was not familiar with the construct and blinded to the original English version, finished the back-translation into English according to the forward-translated document.	No	After comparing the back-translation and the original English version, divergences were identified and discussed with the original author of the MAIA-2.	Yes	A total of 8 interviewees (2 males, 6 females) aged between 19 and 54 years (M = 30.75, SD = 12.18) participated in the cognitive interviews.	Yes	4/5
	Dutch (Netherlands)(Scheffers et al., 2024)	Two forward translations were performed independently by two native Dutch speakers, with proficiency in English and with knowledge of the concept of interoceptive awareness and its meaning in practice.	Yes	The two versions were then compared item-by-item, and discrepancies were resolved.	Yes	Two independent professional bilingual translators, naive to the construct of interoceptive awareness, performed a back-translation into English.	Yes	Discrepancies between the back-translations and the original English version was discussed and resolved	Yes	This resulted in a pre-final Dutch version. In the next step, a pilot study was conducted among 41 respondents to examine the usability and comprehensibility.	Yes	5/5
	Norwegian(Fiskum et al., 2023)	Two independents translators performing separate translations.	Yes	…before discussing and settling any differences in the translations. The Norwegian-translated version was further discussed with two experts in psychosomatic clinical work	Yes	Independent back-translator fluent in Norwegian and English.	No	-	-	-	-	2/5

				and long experience with patient groups with reduced interoception.								
	Persian(Melhi et al., 2021)	A native English speaker and a fluent Persian translator translated Persian into English but had not seen the original version.	Yes	Fifteen experts conducted a qualitative evaluation of content validity, including clinical psychologists, health psychologists, general psychologists, and psychometricians.	Yes	-	-	-	-	Then, 30 participants reviewed and provided feedback on the translated questionnaire to ensure face validity.	Yes	3/5
	Spanish (Peru) (Vivas-Rivas et al., 2025)	A Peruvian translator specialized in psychology, but unfamiliar with the construct, prepared the initial draft of the Spanish translation of the MAIA-2.	No	The lead author (LV-R) then met with a bilingual native Spanish speaker … to read, analyze, and compare the initial draft of the translation with the original MAIA-2 and the Chilean version.	Yes	-	-	-	-	A series of cognitive interviews was conducted to identify and resolve any additional issues with the MAIA-2 items. Eleven adults residing in Peru were contacted.	Yes	2/5
Brief Multidimensional Assessment of Interoceptive Awareness Version 2 (Brief MAIA-2) English (USA)	Polish (Rogowska et al., 2023)	We independently used the forward–backward translation process from English to Polish (and vice versa) by two experts (one was a psychology professor from a Polish university, and the d second was an English native speaker).	No	-	-	We independently used the forward–backward translation process from English to Polish (and vice versa) by two experts (one was a psychology professor from a Polish university, and the d second was an English native speaker).	No	Each item was discussed and corrected if necessary.	Yes	A pilot study was performed on 12 psychology students who participated in a Master's seminary	Yes	2/5
Physical Body Experiences Questionnaire Simplified for Active Aging (PBE-QAG) Italian	English (USA) (Deng et al., 2023)	-	-	-	-	-	-	-	-	-	-	Based on a previous translation
Scale of Body Connection (SBC) English (USA)	Portuguese (Portugal) (Neves et al., 2017)	Two independent translators.	Yes	-	-	These versions were separately backtranslated by a native English speaker.	No	-	-	The final version was pilot-tested with a sample of 15 college students	Yes	2/5
	Portuguese (Portugal) (Price et al., 2017)	-	-	-	-	-	-	-	-	-	-	Based on a previous translation

	Italian(Price et al., 2017)	-	-	-	-	-	-	-	-	-	-	No info
	French (France) (Price et al., 2017)	-	-	-	-	-	-	-	-	-	-	No info
	Dutch (Netherlands) (Price et al., 2017)	-	-	-	-	-	-	-	-	-	-	No info
	English (USA) (Price et al., 2017)	-	-	-	-	-	-	-	-	-	-	No info
	Hebrew (Price et al., 2017)	-	-	-	-	-	-	-	-	-	-	No info
	English (USA) (Cheng et al., 2022)	-	-	-	-	-	-	-	-	-	-	No info
	French (France) (Cheng et al., 2022)	-	-	-	-	-	-	-	-	-	-	No info
	English (Australia) (Cheng et al., 2022)	-	-	-	-	-	-	-	-	-	-	No info
	Dutch (Netherlands) (Cheng et al., 2022)	-	-	-	-	-	-	-	-	-	-	No info
	German(Cheng et al., 2022)	-	-	-	-	-	-	-	-	-	-	No info
	Italian(Morganti et al., 2020)	-	-	-	-	Back-translation procedure by two independent translators.	Yes	Discrepancies emerging from this procedure were discussed until they reached agreement on a common version.	Yes	-	-	2/5

	Spanish (Spain) (del C Quezada-Berumen et al., 2014)	Two native Spanish speakers who were aware of the objective of the scale.	Yes	-	-	Two native English speakers who were not familiar with the SBC then performed a back-translation from Spanish to English.	Yes	Discrepancies between the Spanish and English translators were resolved by agreement.	Yes	-	-	3/5

-, Not indicated
